# Decision Making and Executive Function in Male Adolescents with Early-Onset or Adolescence-Onset Conduct Disorder and Control Subjects

**DOI:** 10.1016/j.biopsych.2009.02.024

**Published:** 2009-07-15

**Authors:** Graeme Fairchild, Stephanie H.M. van Goozen, Sarah J. Stollery, Michael R.F. Aitken, Justin Savage, Simon C. Moore, Ian M. Goodyer

**Affiliations:** a Developmental Psychiatry Section, Department of Psychiatry, Cambridge University, Cambridge, United Kingdom; b Department of Experimental Psychology, Cambridge University, Cambridge, United Kingdom; c School of Psychology, Cardiff University, Cardiff, United Kingdom; d School of Dentistry, Cardiff University, Cardiff, United Kingdom

**Keywords:** Conduct disorder, decision making, developmental taxonomic theory, executive function, reward, risk, stress

## Abstract

**Background:**

Although conduct disorder (CD) is associated with an increased susceptibility to substance use disorders, little is known about decision-making processes or reward mechanisms in CD. This study investigated decision making under varying motivational conditions in CD.

**Methods:**

Performances on the Risky Choice Task (RCT) and the Wisconsin Card Sorting Test (WCST) were assessed in 156 adolescents (84 control subjects, 34 with adolescence-onset CD, and 38 with early-onset CD). The RCT was performed twice, once under normal motivational conditions and once under conditions of increased motivation and psychosocial stress.

**Results:**

Increased motivation and stress led to more cautious decision making and changes in framing effects on the RCT in all groups, although such effects were least pronounced in the early-onset CD group. Participants from both CD subgroups selected the risky choice more frequently than control subjects. Under normal motivational conditions, early-onset CD participants chose the risky choice more frequently in trials occurring after small gains, relative to control subjects and adolescence-onset CD participants. Following adjustment for IQ differences, the groups did not differ significantly in terms of WCST performance.

**Conclusions:**

Differences in decision making between control subjects and individuals with CD suggest that the balance between sensitivity to reward and punishment is shifted in this disorder, particularly the early-onset form. Our data on modulation of decision making according to previous outcomes suggest altered reward mechanisms in early-onset CD. The WCST data suggest that impairments in global executive function do not underlie altered decision making in CD.

Children and adolescents with conduct disorder (CD) (1) show a propensity toward risk taking and reckless behavior, suggesting difficulties with decision making and impulsivity. They are also more susceptible to substance abuse (2), potentially reflecting altered sensitivity of reward mechanisms and persistent selection of options with short-term benefits despite negative long-term consequences.

Decision-making difficulties in CD may stem from deficits in emotional and executive function (EF). Bechara *et al.* (3) have demonstrated that patients with ventromedial frontal cortex damage exhibit decision-making impairments on the Iowa Gambling Task (IGT) (3), interpreting these results as reflecting an inability to use somatic or emotional markers to choose between competing options and, in particular, signify options with potential for adverse consequences (4,5). Conduct disorder is associated with reduced amygdala and anterior insula volume (6) and neuropsychological deficits consistent with putative amygdala dysfunction (7–9). These deficits may alter the influence of emotion on decision making in individuals with CD. Evidence for executive dysfunction in CD or its milder variant, oppositional defiant disorder (ODD), is less compelling, although children with ODD experience difficulty in responding to changes in environmental contingencies, e.g., suppressing responses to previously rewarded stimuli when they become associated with punishment (10).

Given these lines of evidence, decision-making impairments might be expected in individuals with CD, but empirical data are limited. Compared with control subjects, adolescents with disruptive behavior disorders (DBDs) only exhibited deficits in IGT performance when playing it for a second time, a week after the first attempt (11), thereby failing to show improved performance over time.^1^ Iowa Gambling Task performance was significantly impaired in children with high levels of psychopathic traits, relative to control subjects (12). It is not known whether CD participants low in psychopathic traits show decision-making impairments.

The first aim of the study was to characterize decision making under risk, where outcome probabilities are explicitly provided, under differing motivational conditions in male adolescents with CD and healthy control subjects. Iowa Gambling Task performance deficits may reflect impairments in multiple neuropsychological processes, including working memory, reversal learning, or sensitivity to reward/punishment (13). Consequently, we used a modified version of the Risky Choice Task (RCT) (14), a more direct measure of decision making that could be played twice in the test battery to examine effects of increased motivation and stress. We felt it would be informative to measure decision making under conditions of heightened motivation and stress because differences between control subjects and individuals with DBDs may be most evident in “hot” motivational contexts (10,15). This could be partly due to physiological hyporeactivity observed during stress in children and adolescents with DBD, particularly CD and ODD (16,17). Furthermore, by enhancing participants' motivation to perform well on the task, we sought to minimize the possibility that apathy or lack of engagement would underlie group differences in decision making.

A second aim was to assess global executive function in CD using the Wisconsin Card Sorting Test (WCST) (18,19). This would allow us to assess the relationship between global executive function and decision making and test the specificity of changes in motivational or emotional aspects of executive function that would be reflected in RCT performance changes. Among other cognitive processes, the WCST measures set shifting, which can be considered a measure of “cold,” or nonaffective, executive function (20). Neuroimaging studies suggest that the RCT and WCST may recruit partially dissociable neural circuits (21,22), consistent with this fractionation of hot and cold executive function.

Our third aim was to examine potential effects of age of CD onset, as early-onset (or childhood-onset) CD is suggested to be uniquely associated with neuropsychological impairment (23). In contrast, adolescence-onset CD is argued to arise primarily due to social modeling of antisocial peers. We investigated whether this distinction, as suggested by the developmental taxonomic theory (23), would extend to differences in decision making and reward mechanisms. Previous studies reported intact WCST performance in male early-onset and adolescence-onset CD participants (24), although female participants with CD showed increased perseverative errors on the WCST (25). However, few data are available on decision-making processes or hot executive function in CD.

We hypothesized that increased motivation/stress would promote cautious choices on the RCT in control subjects. Adolescents with early-onset CD were predicted to show increased risky decision making relative to control subjects, with differences most pronounced under conditions of increased motivation/stress. We anticipated group differences in experimental gamble frequency following certain outcomes, with CD participants less dissuaded by losses in previous trials due to insensitivity to punishment cues. Given their impaired function, vulnerability to substance abuse, and poorer adult outcomes (26), we also expected heightened risky decision making in adolescence-onset CD participants, contrary to the developmental taxonomic theory (23). Finally, early-onset CD participants were predicted to show increased WCST perseverative errors given previous data showing response perseveration in children with ODD (10) and female adolescents with CD (25).

## Methods and Materials

### Participants

Male adolescents aged 14 years to 18 years were recruited from mainstream schools and colleges, pupil referral units, and the Cambridge Youth Offending Service. All participants gave written informed consent, and the study was approved by the Local Research Ethics Committee.

Diagnostic interviews using the Kiddie Schedule for Affective Disorders and Schizophrenia for School-Age Children-Present and Lifetime Version (K-SADS-PL) (27) were completed with all participants and their caregivers to assess for current and lifetime psychopathology. This process yielded 84 control subjects with no lifetime history of DBD and no current psychiatric illness and 72 adolescents with CD or ODD, of whom 38 had early-onset CD (EO-CD) and 34 had adolescence-onset CD (AO-CD). The latter group included six participants with adolescence-onset ODD only.

Exclusion criteria for participation were IQ < 75 as estimated using Vocabulary and Block Design subtests of the Wechsler Abbreviated Scale of Intelligence (28), presence of pervasive developmental disorder or chronic physical illness, and current use of steroid medication.

Participants were allocated to the EO-CD group if they or their caregivers reported at least one CD symptom and functional impairment was present before the age of 10 or if they met full criteria for ODD before age 10 and developed CD after age 10. Inclusion in the study was based on lifetime diagnoses of CD/ODD, although most (95.8%) index cases had a current CD/ODD diagnosis.

Eleven participants with EO-CD and six with AO-CD had comorbid attention-deficit/hyperactivity disorder (ADHD). All had been medication-free for >6 months. Two AO-CD and five EO-CD participants had comorbid major depressive disorder (MDD).

Psychopathic traits were measured using the Youth Psychopathic Traits Inventory (YPI) (29), and parental socioeconomic status was estimated using the National Statistics Standard Occupational Classification 2000 guidelines. Drug and alcohol use were assessed using the Adolescent Alcohol and Drug Involvement Scale (30).

### Design

The study involved playing the RCT twice: once under normal conditions (no monetary incentive) and then under increased motivation/stress conditions (as part of a standardized laboratory stressor with a monetary incentive, see below). These were played in the same order by all participants.

### Decision-Making Task

**Figure 1:**
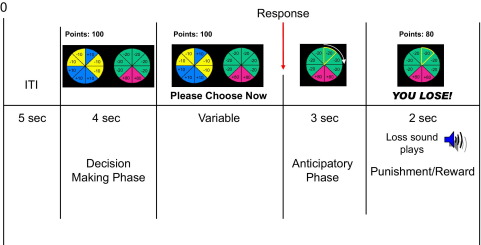
Schematic trial sequence of the modified Risky Choice Task. Available options are shown in a “roulette wheel” format. The control gamble, with an expected value of 0 (.5 × 10 + .5 × −10), is shown on the left, while the experimental gamble, with an expected value of +5 (.75 × −20 + .25 × 80), is shown on the right. Following response selection, a highlight spins around the wheel, gradually becoming slower until it lands on one of the eight wedges. Following this anticipatory phase, verbal and auditory feedback about the outcome (gain or loss) is provided. The revised points total is also displayed (i.e., “Points: 80”).

A modified version of the RCT (14) was used. Two wheels were presented on screen, showing the points available and the relative probability of each outcome. Participants were given 4 seconds to make their choice, after which time “Please choose now” appeared on screen. On most trials, one wheel served as a “control” gamble. It provided a .5 chance of gaining 10 points and a .5 chance of losing 10 points (Figure 1, left wheel). The alternative, “experimental” gamble varied systematically in terms of the probability of a gain (.75 or .25), the magnitude of the possible gain (80 or 20 points), and the magnitude of the possible loss (80 or 20 points) (for example, see Figure 1, right wheel). Different combinations of these variables yielded eight trial types varying in the relative expected value of the two options (delta expected value [ΔEV]) (Supplement 1). For example, in Figure 1, the left wheel has an EV of 0 (.5 × 10 + .5 × −10), whereas the right wheel has an EV of +5 (.25 × 80 + .75 × −20). Thus, the difference in EV (ΔEV) between these choices (in favor of the experimental gamble) is +5.

We also included two trial types in which both options had an equal EV to measure framing effects. The negative framing trial (0 + frame) involved a wheel with a certain gain of 40 points and a wheel with a .5 chance of gaining 80 and a .5 chance of gaining 0 points. The positive framing trial (0 – frame) involved a wheel with a certain loss of 40 points and another wheel with a .5 chance of losing 80 points and a .5 chance of losing 0 points. These trial types were intended to assess effects of decision frame (gaining or losing) on risk aversion. Healthy volunteers show moderate risk aversion (preferring smaller, safer rewards) when comparing possible gains; when comparing possible losses, they show a tendency toward risk seeking (avoiding more certain, but smaller, losses) (31).

All 10 trial types were presented twice per block in a pseudorandom order, and participants played 4 blocks per session. The control and experimental (or risky) gambles appeared randomly on the left or right of the display, and participants indicated their choice using a computer mouse. Participants were given 100 points at the start of each block and were instructed to try to win as many points as possible. Feedback was provided and the revised points total was presented for 2 seconds before the next trial.

### Stress Induction Procedure

Approximately 60 to 75 minutes after lunch, participants were told they would be taking part in a competition with an opponent of a similar age, with a cash prize for the winner. This procedure has been described in detail elsewhere (17). Briefly, it involves inducing frustration and antagonism between the participant and a videotaped opponent. Frustration was induced by having the participant perform a difficult, computer-based manual precision task under time pressure while the opponent and experimenter watched. By design, participants failed to achieve their target score and received negative evaluations of their performance from the opponent. This procedure reliably elicits cortisol secretion and increased autonomic activity in control subjects (16,17). Participants played the RCT for the second time ∼50 minutes after stress onset, at a point when cortisol and heart rate are significantly elevated relative to baseline in control subjects (16).

### Wisconsin Card Sorting Test (WCST)

The WCST assesses the ability to form abstract concepts and shift between response sets (18). The computerized 64-card form of the test (19) was administered under normal motivational conditions.

Following previous research (11), we assessed: 1) number of categories completed, 2) overall trials administered, 3) perseverative errors, 4) nonperseverative errors, 5) trials to complete the first category, and 6) failure to maintain set.

### Data Analysis

Data on demographic and personality characteristics were analyzed using one-way analysis of variance (ANOVA) or chi-square tests. Statistical tests used to analyze RCT and WCST data are described below. Effect sizes are reported as partial eta-squared (η_p_^2^; small ≥ .01, medium ≥ .06, large ≥ .14) (32). Analyses were performed using SPSS 11.5 (SPSS Inc., Chicago, Illinois).

## Results

Table 1 presents demographic information for each group and accompanying statistical analyses.

The groups differed in socioeconomic status (SES) [χ^2^(4) = 23.8, *p* < .001]. Underlying this effect, EO-CD participants were of lower SES than control subjects [χ^2^(2) = 23.7, *p* < .001]. There were fewer nonwhite participants in the EO-CD group than the AO-CD group [χ^2^(1) = 6.0, *p* < .05].

Relative to control subjects, there were more smokers in the AO-CD [χ^2^(1) = 26.3, *p* < .001] and EO-CD groups [χ^2^(1) = 37.7, *p* < .001]. Frequency of alcohol use was higher in the EO-CD group relative to control subjects [χ^2^(1) = 37.7, *p* < .001]. Compared with control subjects, cannabis use was more common in both AO-CD [χ^2^(1) = 15.4, *p* < .001] and EO-CD participants [χ^2^(1) = 19.5, *p* < .001].

### Performance Data

These data were analyzed using a mixed-model ANOVA with group as a between-subjects factor and condition (normal vs. increased motivation/stress) and round (1 to 4) as within-subjects factors. There was no effect of condition (*p* = .87), round (*p* = .67), or group (*p* = .99) on points gained during the task.

### Choice of the Experimental Gamble by Trial Type and Session

#### Effects of Increased Motivation and Stress on Risky Decision Making in Control Subjects

We used a repeated-measures ANOVA with condition (normal vs. increased motivation/stress) and trial type (10 types differing in ΔEV) as within-subjects factors. The dependent variable was the percentage of trials the experimental gamble was chosen in preference to the control gamble.

**Figure 2:**
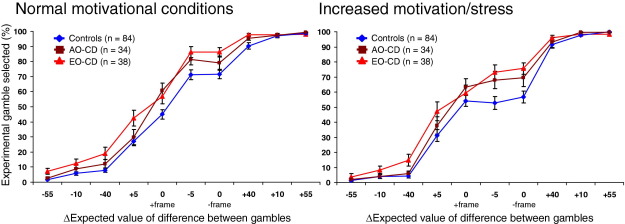
Mean (± SEM) proportion of time the experimental gamble was chosen in preference to the control gamble for each Risky Choice Task trial type, by group. The difference in expected value (ΔEV) between the experimental and control gambles for each trial type is shown along the x axis. Trial types are ordered according to the degree of preference for the riskier experimental gamble observed within the control group under normal motivational conditions. Decision making by all groups was strongly influenced by the experimental contingencies, as shown by the dramatic shift in choice of the experimental gamble across trial types. However, the CD groups were more risky than control subjects, with differences in choice of the experimental gamble over the control gamble most apparent in the middle of the decision-making curve. EO-CD participants were still susceptible to this framing effect when playing under conditions of increased motivation/stress. AO-CD, adolescence-onset conduct disorder; CD, conduct disorder; EO-CD, early-onset conduct disorder; ΔEV, difference in expected value.

Figure 2 shows that control subjects chose the experimental gamble less frequently under conditions of increased motivation/stress (main effect of condition [*F*(1,83) = 5.77, *p* < .05, η_p_^2^ = .07].

There was a main effect of trial type [*F*(4.02,333.66) = 361.09, *p* < .0001, η_p_^2^ = .81] and a trial type × condition interaction [*F*(5.40,448.00) = 17.43, *p* < .001, η_p_^2^ = .17]. This interaction was largely driven by changes in decision making on the framing trials. Control subjects showed a framing effect under normal conditions in favor of increased choice of the experimental gamble on the negatively framed trial (0 – frame), relative to the positively framed trial (0 + frame). This effect of decision frame was attenuated under conditions of increased motivation/stress.

### Group Comparisons

Data were available for 156 participants (84 control subjects, 34 AO-CD, and 38 EO-CD) under both normal and increased motivation/stress conditions (Figure 2). These data were analyzed as above, with the addition of group as a between-subjects factor in a mixed-model ANOVA.

There were effects of trial type [*F*(4.18,639.17) = 597.10, *p* < .0001, η_p_^2^ = .80], condition [*F*(1,153) = 11.34, *p* < .001, η_p_^2^ = .07], and group [*F*(2,153) = 11.08, *p* < .001, η_p_^2^ = .13] on experimental gamble choice. Post hoc group comparisons showed that both CD subgroups chose the experimental gamble more frequently than control subjects (*p* < .05 and *p* < .001 for the AO-CD and EO-CD groups, respectively), but there was no difference between CD subgroups (*p* = .27). Post hoc comparison of conditions indicated that participants selected the experimental gamble less frequently under conditions of increased motivation/stress.

In addition to these effects, there were significant trial type × group [*F*(8.00,612.44) = 2.31, *p* = .019, η_p_^2^ = .03] and trial type × condition [*F*(6.08,930.47) = 16.65, *p* < .001, η_p_^2^ = .10] interactions. The trial type × group interaction was investigated by assessing group effects for each trial type independently. These analyses revealed that CD participants' tendency to select the experimental gamble more frequently than control subjects was significant only on trial types with a ΔEV of −40, +5, −5, and on the negatively framed trial (0 – frame). Similar analyses of the trial type × condition interaction showed that participants selected the experimental gamble significantly less frequently under increased motivation/stress conditions only on trial types with a ΔEV of −40 and −5 and on the negatively framed trial. In contrast, they selected the experimental gamble significantly more frequently on the positively framed trial when under increased motivation/stress.

#### Gamble Frequency Following Trials in Which the Experimental Gamble Was Chosen

For each participant, four types of gamble ratio were calculated: the percentage of time the experimental gamble was chosen in a trial immediately following either a large loss (−80), a small loss (−20), a large gain (+80), or a small gain (+20). Data from four control subjects were excluded due to floor effects (they never gambled when large losses were available when playing under conditions of increased motivation/stress). We ran a mixed-model ANOVA with group as a between-subjects factor and condition, outcome (gain vs. loss), and magnitude (large vs. small) as within-subjects factors.

There were effects of condition [*F*(1,149) = 6.48, *p* = .012, η_p_^2^ = .04], group [*F*(2,149) = 7.42, *p* = .001, η_p_^2^ = .09], and outcome [*F*(1,149) = 8.42, *p* < .005, η_p_^2^ = .05] on gamble frequency. Underlying the condition effect, participants overall selected the experimental gamble less frequently after a gamble trial in the increased motivation/stress condition than in the normal motivation condition. Post hoc group comparisons showed that the EO-CD group selected the experimental gamble more frequently after a gamble trial than the control (*p* < .001) and AO-CD groups (*p* < .05); the control and AO-CD groups did not differ (*p* = .60). Underlying the outcome effect, participants chose the experimental gamble more frequently in trials occurring after losses than gains.

There was also a four-way condition × group × outcome × magnitude interaction [*F*(2,149) = 3.37, *p* < .05, η_p_^2^ = .04], leading us to explore data from the normal and increased motivation/stress conditions independently.

**Figure 3:**
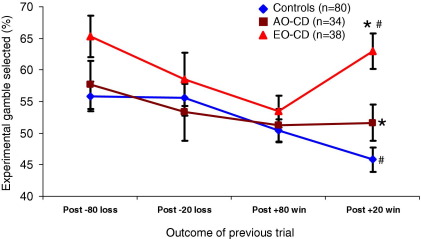
Mean (± SEM) percentage of trials in which the experimental gamble was chosen over the control gamble, after specific outcomes in the previous trial, by group. This shows experimental gamble choice under normal motivational conditions only. Participants with early-onset CD were significantly more likely than the other two groups to select the experimental gamble in trials occurring after a small gain (+20). *p < .05; #*p* < .001. AO-CD, adolescence-onset conduct disorder; EO-CD, early-onset conduct disorder.

Analysis of the normal motivation condition using a mixed-model ANOVA (3 × 2 × 2) revealed effects of group [*F*(2,149) = 4.97, *p* < .01, η_p_^2^ = .06] and outcome [*F*(1,149) = 11.09, *p* < .001, η_p_^2^ = .07] and a three-way group × outcome × magnitude interaction [*F*(2,149) = 4.82, *p* = .01, η_p_^2^ = .06]. This interaction was driven by more frequent gambling after a small gain in the EO-CD group, relative to the other groups [*F*(2,151) = 12.8, *p* < .001; post hoc: control vs. EO-CD, *p* < .001; AO-CD vs. EO-CD, *p* < .05] (Figure 3). Post hoc comparison on the effect of outcome showed that participants gambled more frequently after losses than gains (*p* = .005). Post hoc group comparisons showed that EO-CD participants gambled more frequently than control subjects (*p* < .01).

A mixed-model ANOVA using data from the increased motivation/stress condition revealed an effect of group [*F*(2,149) = 5.64, *p* < .005, η_p_^2^ = .07] but no other effects. Post hoc group comparisons showed that EO-CD participants gambled more frequently than control subjects (*p* < .005).

### Confounding Factors

Using analysis of covariance (ANCOVA), we examined whether our group effects could be explained by group differences in IQ, YPI (psychopathic traits) score, SES, ethnicity, and cigarette, alcohol, or cannabis use. In analyzing the RCT data, the only significant covariate was YPI score (*p* < .05). In an ANCOVA model including this factor, the group effect remained highly significant (*p* = .005). To explore this effect further, we divided the CD participants into high and low psychopathy groups using a cutoff of 2.5 on the YPI (33) and repeated the analyses using these subgroups. There was no clear effect of variation in psychopathic traits on decision making in either motivational condition (Supplement 2). Youth Psychopathic Traits Inventory score was also the only significant covariate (*p* < .05) in the analyses of experimental gamble frequency following a gamble trial, but again, group effects and interactions remained significant in the model including this covariate.

To verify that the group effects reported did not arise solely through differences in estimated IQ, we repeated our analyses excluding control subjects with IQ > 110 and EO-CD participants with IQ < 80 (to equate groups on this variable, *p* > .2). All group differences and interactions remained significant (Supplement 3).

Since the normal and increased motivation/stress conditions were always played in the same order, effects of stress were necessarily confounded with both order and motivational differences. To assess the effect of factors other than stress, we used a small (*n* = 10) sample of control subjects, recruited in the same way as control subjects in the main study. They performed the RCT twice, with an equivalent change in motivational context (payment for good performance) the second time but without the social stress manipulation. There was a similar overall pattern in decision-making performance but no significant differences between conditions. The nonsignificant tendency (*F* < 1) was toward increased gamble frequency in the second, increased motivational condition, the reverse pattern to that seen with a stress manipulation. This suggests that the differences between conditions in the main experiment may be best described as effects of a stress manipulation.

Detailed examination of the data relating to gamble frequency following a gamble trial did not reveal any sequence effects that could have biased the main group effects. The 10 trial types were presented in a pseudorandom fashion and there were no systematic differences between groups in terms of the trial types that occurred following a specific event (e.g., a small gain).

### Wisconsin Card Sorting Test

Data were unavailable for one control, two AO-CD, and two EO-CD participants. Apart from two EO-CD participants, all participants completed the six categories. The data were log-transformed to correct for a nonnormal distribution. Estimated IQ was a significant covariate in these analyses; thus, we included IQ as a covariate in separate ANCOVAs, assessing group effects on each WCST variable.

Following adjustment for group differences in IQ, the groups did not significantly differ on categories completed, overall trials administered, perseverative errors, nonperseverative errors, trials to first category, or failure to maintain set. The IQ-adjusted mean scores for each group are shown in Table 2.

Given that our experimental design involved sampling from intact groups that may have been predicted to differ in IQ (34) and because there are difficulties in interpreting ANCOVA-adjusted means (35), we report nonadjusted WCST data and accompanying one-way ANOVA results in Supplement 4.

## Discussion

Our key findings were: 1) increased motivation/stress led to a more cautious pattern of decision making in both control subjects and CD participants; 2) increased motivation/stress affected control participants' choices in framing trials, such that the effect of the decision frame (gaining or losing) was markedly reduced; 3) both CD groups selected the experimental gamble (the risky choice) on the RCT more frequently than control subjects, with such differences most apparent on framing trials and those in which the difference in expected value between choices was relatively small; 4) there were group differences in reward sensitivity, with early-onset CD participants selecting the experimental gamble more frequently in trials following a small gain than the other groups; and 5) such changes in decision making under risk did not appear to be explained by deficits in global, or cold, executive function in CD (since there were no group differences in WCST performance following IQ adjustment).

The present study demonstrated that early-onset CD participants selected more risky choices than control subjects under both normal motivation and increased motivation/stress conditions. The latter observation suggests that apathy or lack of engagement with the task is unlikely to explain the observed group differences in decision making. Similar to control subjects, the early-onset CD participants' choices were strongly influenced by task contingencies, but they showed increased risk taking across all trial types, suggesting a criterion shift in decision-making behavior. It is currently unclear whether this shift reflects increased sensitivity to reward or reduced sensitivity to loss or a combination of these factors. In contrast with control subjects, they showed framing effects in both the normal motivation and increased motivation/stress conditions. This may be related to an attenuation of physiological changes occurring under stress in this group (16,17) or to a reduced awareness of such changes (impaired interoception). Interestingly, while receipt of a small gain suppressed gambling in control subjects, it appeared to promote gambling in the early-onset CD group. One interpretation of this finding is that increased reinforcement or reward may be required to satisfy individuals with early-onset CD and hence suppress their risky decision making.

The adolescence-onset CD group also showed increased choice of the experimental gamble relative to control subjects under both motivational conditions. As noted above, their decision making was modulated by previous trial outcome in a similar manner to control subjects, i.e., they showed no evidence for altered reward sensitivity.

Following adjustment for differences in IQ, there were no group differences on the WCST. In addition, virtually all participants completed the test. This suggests that global or cold executive function is broadly intact in CD, consistent with earlier work (24) but at variance with our prediction of increased WCST perseverative errors in early-onset CD participants. As such, it seems unlikely that deficits in global EF underlie the observed group differences in decision making or reward sensitivity.

Considered together, these data provide partial support for the developmental taxonomic theory of antisocial behavior. There were clear differences in decision-making behavior and reward sensitivity between control subjects and participants with early-onset CD. Differences in decision making between control and adolescence-onset CD participants were not predicted by the theory, however. A tendency toward increased risk taking may partly explain why individuals with adolescence-onset CD have a negative prognosis. Interestingly, the CD subgroups only differed significantly in terms of reward sensitivity.

Two limitations are noted. First, differences in risky decision making may reflect reduced sensitivity to rewarding or punishing outcomes, increased risk taking (preferring gambles with more uncertain outcomes because they generate increased arousal), or other motivational differences. Additional research is needed to disaggregate these factors. Second, the condition effects on RCT performance that we have attributed to stress/increased motivation could instead be due to learning or order effects. This limitation could be overcome using a counterbalanced, crossover design. However, RCT performance did not differ between normal and increased motivation conditions in a subgroup of control subjects not exposed to stress.

### Conclusions

This study demonstrated that increased motivation/stress attenuated risky decision making in healthy adolescents and reduced their susceptibility to framing effects. Both CD subgroups showed increased risky decision making across motivational conditions relative to control subjects, although differences were most marked for the early-onset CD group. The latter group was also more likely to select the experimental gamble after receiving a small gain compared with the other groups, potentially suggesting differences in reward sensitivity. Such alterations in reward mechanisms could increase vulnerability to substance dependence and pathological gambling.

## Figures and Tables

**Table 1:** Participant Characteristics

Measure	CON (*n* = 84)		AO-CD (*n* = 34)		EO-CD (*n* = 38)		*p*	Post Hoc
	Mean	SD	Mean	SD	Mean	SD		
Age (years)	15.77	.82	15.54	.90	15.75	.75	.38	
Estimated IQ	105.92	12.17	98.29	11.32	93.00	10.60	<.001	CON > AO, EO
Psychopathic Traits (YPI)	2.10	.30	2.38	.27	2.45	.35	<.001	CON < AO, EO
CD Symptoms	.24	.63	5.44	2.72	8.32	3.17	<.001	CON < AO < EO
	*n*	%	*n*	%	*n*	%		
Socioeconomic Status								
Low	10	11.9	8	23.5	16	42.1		
Middle	15	17.9	7	20.6	10	26.3		
High	54	64.3	14	41.2	7	18.4	<.001	
Ethnicity								
Caucasian	77	91.7	29	85.3	38	100		
Nonwhite	7	8.3	5	14.7				
Frequent/Daily Use of								
Tobacco	14	16.7	22	64.7	28	73.7		
Alcohol	3	3.6	2	5.9	9	23.7		
Cannabis	7	8.3	13	38.2	16	42.1		

Socioeconomic status information was unavailable for five CON, five AO-CD, and five EO-CD participants. AO, adolescence onset; AO-CD, adolescence-onset conduct disorder, CD, conduct disorder; CON, control; EO, early onset; EO-CD, early-onset conduct disorder; IQ, intelligence quotient; YPI, Youth Psychopathic Traits Inventory.

**Table 2:** IQ-Adjusted Wisconsin Card Sorting Test (WCST) Mean Scores for Control, Adolescence-Onset CD, and Early-Onset CD Groups and ANCOVA Results Including IQ As a Covariate

Measures	CON (*n* = 83)		AO-CD (*n* = 32)		EO-CD (*n* = 36)		*p* Value
	Mean	SE	Mean	SE	Mean	SE	
Categories completed	6.0	.0	6.0	.0	5.9	.0	.18
Trials administered	84.9	1.5	86.0	2.3	88.3	2.3	.58
Perseverative errors	7.5	.4	8.3	.6	9.2	.6	.22
Nonperseverative errors	7.9	.5	7.2	.8	8.6	.8	.72
Trials to first category	12.4	.4	11.7	.7	12.5	.7	.56
Failure to maintain set	.4	.1	.4	.1	.3	.1	.81

Note that WCST data were unavailable for one CON, two AO-CD, and two EO-CD participants. ANCOVA, analysis of covariance; AO-CD, adolescence-onset conduct disorder; CD, conduct disorder; CON, control; EO-CD, early-onset conduct disorder; IQ, intelligence quotient; WCST, Wisconsin Card Sorting Test.

## Footnotes

1 It should be noted that the DBD group in that study was largely comprised of participants with attention-deficit/hyperactivity disorder (only 5 of 33 participants had CD); thus, it is not clear whether these findings can be generalized to adolescents with CD/ODD. Furthermore, the experimental groups were unbalanced in terms of gender, with more male participants in the DBD group relative to the control group.
