# Supplementary material for: Decision Making and Executive Function in Male Adolescents with Early-Onset or Adolescence-Onset Conduct Disorder and Control Subjects
Source: Biol Psychiatry. 2009 Jul 15;66(2):162–8. doi: 10.1016/j.biopsych.2009.02.024 (PMC2733860; doi:10.1016/j.biopsych.2009.02.024)
Supplement: Supplement 1 [file mmc1.pdf]

Supplementary Table 1: The ten trial types included in the Risky Choice Task, including the balanced ‘framing’ trials in which the expected value of each wheel is the same, but one wheel has a certain outcome (either +40 or -40) and one has an uncertain outcome (a .5 chance of either winning or losing 80, and a .5 chance of receiving 0 points). Pr(Gain) indicates the probability of a gain.

Experimental gamble			Control gamble			Difference in expected value (ΔEV) between gambles
Pr(Gain)	Gain	Loss	Pr(Gain)	Gain	Loss	
0.25	20	-80	0.50	10	-10	-55
0.25	80	-80	0.50	10	-10	-40
0.25	20	-20	0.50	10	-10	-10
0.75	20	-80	0.50	10	-10	-5
0.25	80	-20	0.50	10	-10	+5
0.75	20	-20	0.50	10	-10	+10
0.75	80	-80	0.50	10	-10	+40
0.75	80	-20	0.50	10	-10	+55
0.50	0	-80	0.00	0	-40	0 (- frame)
0.50	80	0	1.00	40	0	0 (+ frame)
