# Supplementary material for: Decision Making and Executive Function in Male Adolescents with Early-Onset or Adolescence-Onset Conduct Disorder and Control Subjects
Source: Biol Psychiatry. 2009 Jul 15;66(2):162–8. doi: 10.1016/j.biopsych.2009.02.024 (PMC2733860; doi:10.1016/j.biopsych.2009.02.024)
Supplement: Supplement 2 [file mmc2.pdf]

Supplementary Figure 1

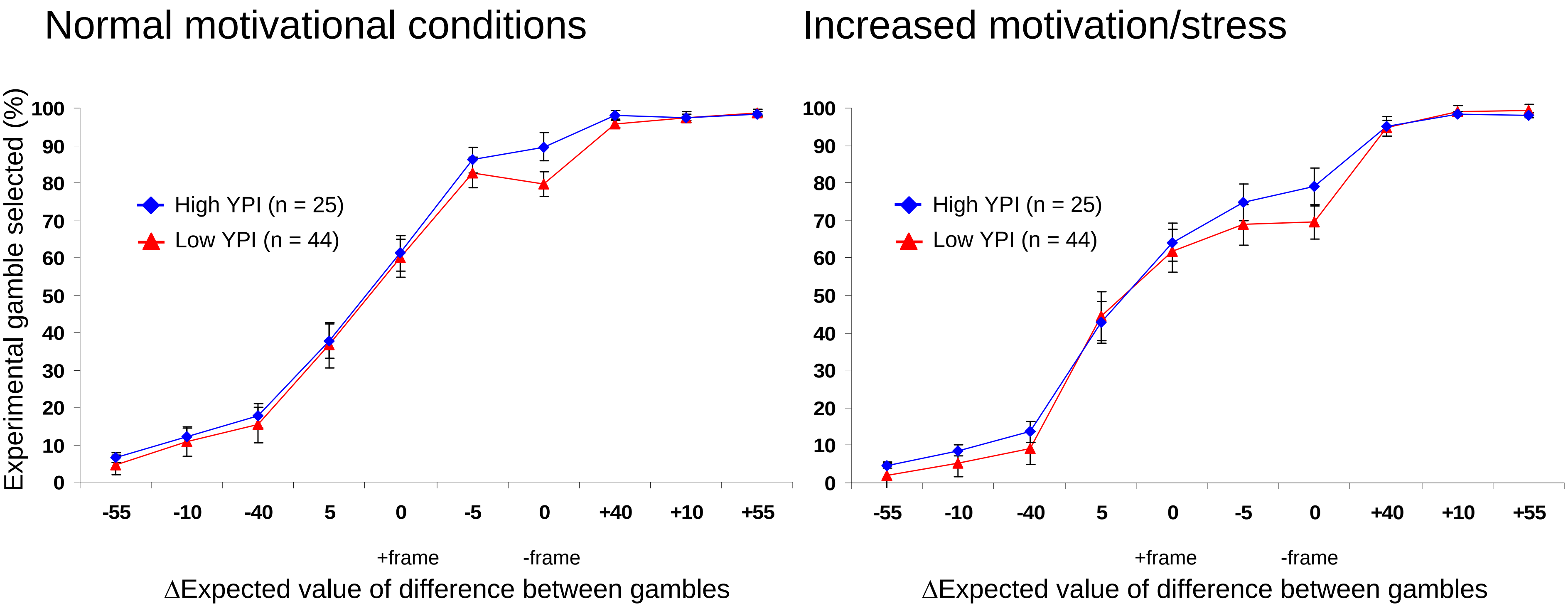

Supplementary Figure 1. Mean (± SEM) proportion of time the experimental gamble was chosen in preference to the control gamble for each trial type, in participants with Conduct Disorder (CD) only. Instead of using the early-onset versus adolescence-onset CD distinction, the total CD group is split according to self-reported psychopathic traits into high and low psychopathy groups (High and Low YPI, respectively). The difference in expected value (ΔEV) between the experimental and control gambles for each trial type is shown along the X-axis. Trial types are ordered according to the degree of preference for the riskier experimental gamble observed within the control group. The left panel shows performance under normal motivational conditions, whereas the right panel shows task performance under conditions of increased motivation and stress. As can be seen, although both low and high psychopathy groups showed reduced risky decision-making under increased motivation/stress conditions relative to normal conditions, there were no clear differences between these groups. Thus, while individuals with CD differ from controls, particularly if they have the early-onset form of the disorder, individuals with CD and high levels of psychopathic traits do not appear to show increased risky decision-making on this task relative to those with CD and lower levels of such personality traits. YPI, Youth Psychopathic traits Inventory.
