# Supplementary material for: Decision Making and Executive Function in Male Adolescents with Early-Onset or Adolescence-Onset Conduct Disorder and Control Subjects
Source: Biol Psychiatry. 2009 Jul 15;66(2):162–8. doi: 10.1016/j.biopsych.2009.02.024 (PMC2733860; doi:10.1016/j.biopsych.2009.02.024)
Supplement: Supplement 3 [file mmc3.pdf]

Supplementary Figure 2

Normal motivational conditions

Increased motivation/stress

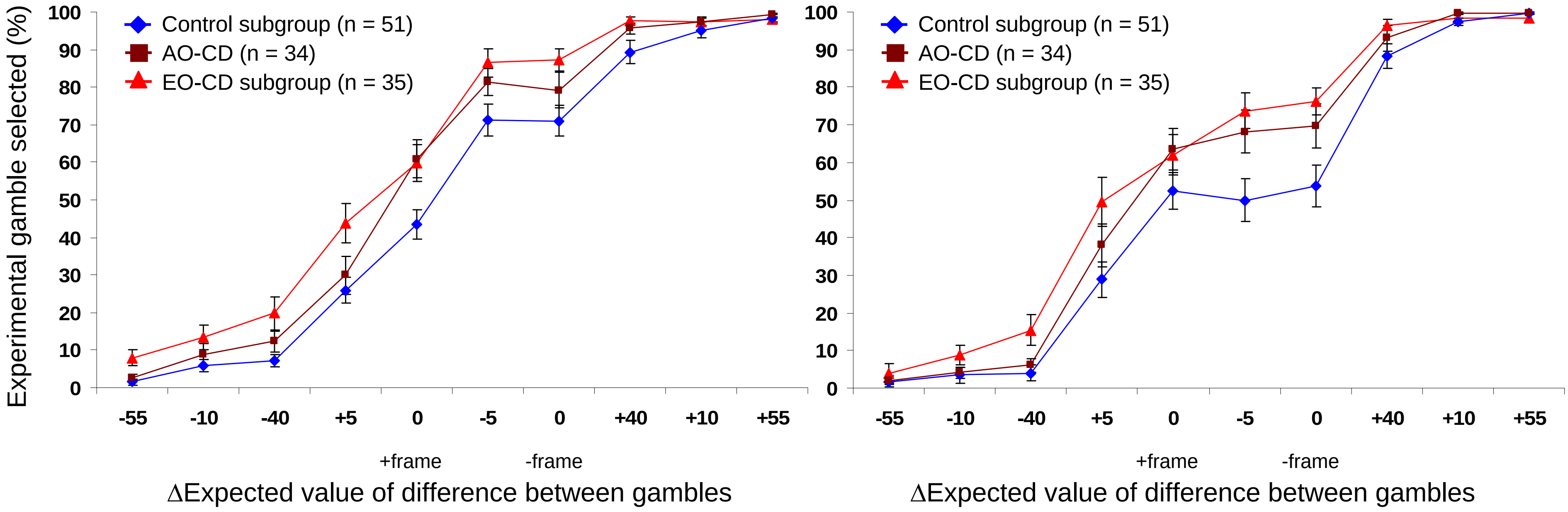

Supplementary Figure 2. Mean (± SEM) proportion of time the experimental gamble was chosen in preference to the control gamble for each trial type, by group, in a subset of participants who were matched on estimated IQ. The difference in expected value (ΔEV) between the experimental and control gambles for each trial type is shown along the X-axis. Trial types are ordered according to the degree of preference for the riskier experimental gamble observed within the control group under normal motivational conditions. The left panel shows performance under normal motivational conditions, whereas the right panel shows performance under conditions of increased motivation and stress. This figure shows that the group differences in risky decision-making reported in the text of the main article and illustrated in Figure 2 are not due to differences in estimated IQ, since the Risky Choice Task performances were virtually identical in this subset of IQ-matched participants, relative to the larger cohort. AO-CD, adolescence-onset Conduct Disorder; EO-CD, early-onset Conduct Disorder.
