# Supplementary material for: Decision Making and Executive Function in Male Adolescents with Early-Onset or Adolescence-Onset Conduct Disorder and Control Subjects
Source: Biol Psychiatry. 2009 Jul 15;66(2):162–8. doi: 10.1016/j.biopsych.2009.02.024 (PMC2733860; doi:10.1016/j.biopsych.2009.02.024)
Supplement: Supplement 4 [file mmc4.pdf]

Supplementary Table 2: Raw mean scores on the Wisconsin Card Sorting Test (WCST) for control, adolescence-onset CD and early-onset CD groups and accompanying ANOVA results, prior to correction for between-group differences in IQ.

**Measures**	**CON (n = 83)**		**AO-CD (n = 32)**		**EO-CD (n = 36)**			
	**Mean**	**SD**	**Mean**	**SD**	**Mean**	**SD**	**P**	**Post-hoc**
**Wisconsin Card Sorting Test**								
**Categories completed**	6.0	0.0	6.0	0.0	5.9	0.5	<.05	No differences
**Trials administered**	83.6	12.0	86.8	12.8	90.6	16.2	<.05	CON < EO-CD
**Perseverative errors**	7.2	2.7	8.4	4.0	9.7	4.9	<.005	CON < EO-CD
**Non-perseverative errors**	7.5	4.7	7.4	3.9	9.4	5.5	.16	
**Trials to 1st category**	12.4	4.1	11.7	2.3	12.4	4.2	.54	
**Failure to maintain set**	0.4	0.7	0.5	0.7	0.4	0.6	.84	

Note that WCST data were unavailable for one CON, two AO-CD, and two EO-CD participants.
Key: CON, control; AO-CD, adolescence-onset Conduct Disorder, EO-CD, early-onset Conduct Disorder.
